# The efficacy of combined antidepressant therapy in depressed patients

**DOI:** 10.1192/j.eurpsy.2025.1348

**Published:** 2025-08-26

**Authors:** Z. Mitic, A. Spasovska Trajanovska

**Affiliations:** 1 Psychiatry Practice “Zora Mitic”; 2 Psychiatry Hospital Skopje, Skopje, North Macedonia

## Abstract

**Introduction:**

According to certain researches, it is known that depressed patients are represented by a high percentage of psychiatric diseases. In these patients, there is a reduction of volitional dynamism, especially the drive to live as well as the social drive.

Failure to treat these patients can lead to serious consequences, suicides and social dysfunction (reluctance for any activity and social isolation).

That is why it is of great importance to apply adequate antidepressant therapy. On the other hand, according to data from the literature, it is known that depression abounds with a multitude of symptoms that cannot be overcome with the use of only one type of antidepressant, so a combination of them is needed.

**Objectives:**

The Aim of this study is to assess the effectiveness of combined antidepressant therapy in the treatment of depressed patients

**Methods:**

This prospective present study included groups of 30 patients of either sex between 26-55 years with diagnosis F32, F33 evaluated in Private Psychiatric Institution Zora Mitic, Skopje. The study was conducted for 6 month. All the patients was written informed consent. Exclusion criteria was exist depressive disorders, patients with another psychiatric disorders and another organic disorders were not included in the study. The patients were examination before treatment and after treatment with Mirtazepine 15-30mg/day and Sertraline 50-100mg/day doses. The patients were assess using sociodemographic information by semi-structured questionnaire specially designed for the study. The sociodemographic data was:, marital status, education status and employment. Depression in patients was assessed by HAMD scale: 21 items graded ranging 0-4. The results obtained were compared using the t-test and Chi-square test. The quantitative data was expressed in number and percentage. The p value of statistical significance was set at p<0.05

**Results:**

Results The obtained results indicated that there is a statistical difference in depressive patients before treatment and after six months of treatment with antidepressant therapy the level of education (p=0,07), marital status (p=0,12); employment (p=0, 09) were without statistical significantly. But we got that the HAMD scale score were statistically significant when compare before treatment and after treatment with antidepressant therapy (p=0,03).

**Conclusions:**

ConclusionThe results obtained in the study confirmed that the combined antidepressant therapy in patients with rich symptomatology reduced the depressive symptomatology and enabled an improvement in the functioning of the individuals.

**Disclosure of Interest:**

None Declared

